# Distinct Patterns of Interhemispheric Connectivity in Patients With Early- and Late-Onset Alzheimer’s Disease

**DOI:** 10.3389/fnagi.2018.00261

**Published:** 2018-09-06

**Authors:** Kai-Cheng Li, Xiao Luo, Qing-Ze Zeng, Xiao-Jun Xu, Pei-Yu Huang, Zhu-Jing Shen, Jing-Jing Xu, Jiong Zhou, Min-Ming Zhang

**Affiliations:** ^1^Department of Radiology, The Second Affiliated Hospital of Zhejiang University School of Medicine, Hangzhou, China; ^2^Department of Neurology, The Second Affiliated Hospital of Zhejiang University School of Medicine, Hangzhou, China

**Keywords:** early-onset Alzheimer’s disease, resting-state functional MRI, voxel-mirrored homotopic connectivity, diffusion tensor imaging, corpus callosum, gray matter volume

## Abstract

**Background**: Early-onset Alzheimer’s disease (EOAD) presents a different clinical profile than late-onset Alzheimer’s disease (LOAD). Neuroimaging studies have demonstrated that patients with EOAD present more atrophy and functional disconnection than LOAD patients. However, it remains unknown whether the interhemispheric functional disconnection or its underlying structural impairment contributes to the different clinical profiles of EOAD and LOAD.

**Methods**: According to the arbitrary cut-off age of 65, we included 22 EOAD patients, 27 LOAD patients and 38 healthy controls (further divided into 21 relatively young and 17 old controls). Participants underwent resting-state functional MRI, diffusion tensor imaging (DTI) and comprehensive neuropsychological assessments. We used voxel-mirrored homotopic connectivity (VMHC) to examine interhemispheric functional connectivity. Then, we calculated the diffusion index based on tract-based spatial statistics (TBSS). Two-sample *t*-tests were used to assess the interhemispheric connectivity differences between each patient group and its corresponding control group.

**Results**: We found that the EOAD patients had lower VMHC in the hippocampus, parahippocampal gyrus (PHG), superior temporal gyrus (STG) and inferior parietal cortex (IPC) than did controls. Consistently, the EOAD patients exhibited white matter (WM) tract impairment in the posterior regions. On the other hand, the LOAD patients displayed increased VMHC and impaired WM tracts in the frontal region. Correlation analyses showed that VMHC in the IPC was related to executive function in the EOAD patients (*r* = −0.67, *P* < 0.05).

**Conclusion**: In contrast to the LOAD patients, patients with EOAD exhibited more widely disrupted interhemispheric functional and structural connectivity, which overlapped well across brain regions. In addition, for the EOAD patients, decreased interhemispheric connectivity related to executive deficits. Our study suggested that different interhemispheric connectivity damage patterns may contribute to the distinct clinical profiles in EOAD and LOAD.

## Introduction

As the most common form of dementia, Alzheimer’s disease (AD) is characterized by memory and other cognitive ability deficits. According to the arbitrary age cut-off of 65 years, we can further classify AD into early-onset AD (EOAD) and late-onset AD (LOAD; McKhann et al., 1984; Filley et al., 1986). Although they share the same neuropathological hallmarks, EOAD features different clinical characteristics from LOAD. Specifically, EOAD patients have worse attention, executive function, language and visuospatial function, but less memory impairment (Karas et al., 2007; Stopford et al., 2008; Smits et al., 2012; Joubert et al., 2016). Moreover, patients with EOAD have a more aggressive disease course and more psychiatric problems than LOAD patients (Koss et al., 1996; Toyota et al., 2007; Koedam et al., 2010). Until now, the underlying mechanisms have remained unclear.

AD has been considered a disconnection syndrome, to some extent, that features a potential decrease in interhemispheric connectivity (Lakmache et al., 1998; Delbeuck et al., 2003; Bartzokis et al., 2004; Liu et al., 2014). Notably, a previous functional MRI study demonstrated that AD patients could perform normally in intrahemispheric-based tasks while performing poorly on tasks requiring interhemispheric communication (Lakmache et al., 1998). This phenomenon is supported by morphologic studies documenting corpus callosum (CC) atrophy in AD patients (Wang et al., 2006; Chaim et al., 2007). Furthermore, microstructural studies demonstrated that EOAD patients have a widespread pattern of white matter (WM) loss, especially in the CC splenium and dorsal temporoparietal regions (Canu et al., 2012). In contrast, WM loss in LOAD patients is mainly confined to memory-related areas (Canu et al., 2012). Building on these studies, we therefore hypothesized that different clinical symptoms between EOAD and LOAD patients might be attributed to distinct deficits in interhemispheric functional or structural connectivity.

Voxel-mirrored homotopic connectivity (VMHC) assesses interhemispheric resting-state functional connectivity (RSFC) by quantifying the RSFC in one hemisphere and its mirrored counterpart in the other. The approach has been well validated in previous studies with AD patients (Wang et al., 2015; Luo et al., 2016), individuals with a high risk of developing AD (Luo et al., 2016), and healthy aging individuals (Zuo et al., 2010). Specifically, these studies proved that interhemispheric connectivity calculated by VMHC can adequately reflect the functional consequences of pathologies. Furthermore, we examined WM connectivity by diffusion tensor imaging (DTI), based on the hypothesis that there is a strong link between structural and functional connectivity. Diffusion indices, including fractional anisotropy (FA), mean diffusivity (MD), axial diffusivity (AxD) and radial diffusivity (RD), were used to assess interhemispheric structural connectivity (Liu et al., 2011; Li et al., 2013). Currently, based on multi-modal MRI imaging methods, we aimed to test the hypothesis that patients with EOAD and LOAD have different damage patterns of interhemispheric connectivity. Moreover, we sought to clarify the relationships between neuroimaging indices and cognitive performance.

## Materials and Methods

### Subjects

We recruited patients from the Second Affiliated Hospital of Zhejiang University School of Medicine. The Medical Ethics Committee of the Second Affiliated Hospital, Zhejiang University School of Medicine, approved this study, and all participants provided informed consent at the beginning of the study. The diagnosis of early AD was made by an experienced neurologist (JZ) according to the NINCDS-ADRDA Alzheimer’s Criteria for “probable AD” (McKhann et al., 1984). The cut-off of 65 years of age was used to distinguish EOAD from LOAD, primarily established on clinical grounds (Amaducci et al., 1986; Filley et al., 1986; Kim et al., 2005). The neurologist confirmed the age of onset according to the information given by the patient and caregiver. The inclusion criteria for AD included: (a) having an MMSE scores between 20 and 26 (inclusive); (b) having a clinical dementia rating (CDR) of 0.5 or 1.0; and (c) satisfying the NINCDS/ADRDA criteria for probable AD (McKhann et al., 1984). Normal controls (NC) consisted of family members of patients and volunteers recruited through advertisements posted in the hospital. The inclusion criteria for NC the following: (a) having an MMSE between 24 and 30 (inclusive); (b) having a CDR score of 0; (c) having a normal Wechsler Memory Scale Logical Memory (WMS-LM) delay score (in detail: ≥9 for subjects with 16 or more years of education; ≥5 for subjects with 8–15 years of education; and ≥3 for 0–7 years of education); (d) non-clinical depression (geriatric depression scale, GDS score <5); and (e) non-demented. We excluded subjects with the following manifestations: (1) significant medical, neurological (other than probable AD), or psychiatric illness; (2) a history of obvious head trauma; (3) use of non-AD-related medication known to influence cerebral function; (4) signs of clinical depression (GDS score ≥5); and (5) alcohol or drug abuse; and (6) left-handedness.

In the end, we included 24 EOAD and 27 LOAD right-handed patients. Given the significant difference in age between the EOAD and LOAD patients, 21 young controls and 17 old controls were included to match the EOAD and LOAD patients, respectively. Table 1 shows the details of the demographic characteristics.

**Table 1:** Demographics and clinical characteristics.

Variables	EOAD patients (*n* = 22)	Young controls (*n* = 21)	EOAD vs. Young controls (*p* value)	LOAD patients (*n* = 27)	Old controls (*n* = 17)	LOAD vs. Old controls (*p* value)	EOAD vs. LOAD (*p* value)
Age (years)	61.50 ± 4.55	60.81 ± 2.82	0.55	75.04 ± 3.90	72.82 ± 4.23	0.08	<0.001*
Education (years)	9.91 ± 2.86	10.91 ± 3.90	0.35	11.96 ± 3.71	10.01 ± 4.51	0.13	0.04*
Gender (females/males)	16/6	13/8	0.33	14/13	9/8	0.60	0.12
GDS	1.29 ± 0.90	1.33 ± 1.32	0.89	1.30 ± 1.07	1.55 ± 1.29	0.54	0.97
MMSE	21.81 ± 2.38	27.81 ± 1.66	<0.001*	21.11 ± 2.71	27.81 ± 1.80	<0.001*	0.36
MoCA	15.48 ± 3.68	24.19 ± 2.89	<0.001*	15.31 ± 3.40	23.23 ± 3.87	<0.001*	0.87

Data are presented as the means ± standard deviations. EOAD, early-onset Alzheimer’s disease; LOAD, late-onset Alzheimer’s disease; GDS, geriatric depression scale; MMSE, Mini-Mental State Examination; MoCA, Montreal Cognitive Assessment scale. *p < 0.05, significant difference.

### Neuropsychological Tests

All patients underwent extensive neuropsychological testing, including assessment of general cognition (Mini-Mental State Examination, MMSE; Montreal Cognitive Assessment scale, MoCA) and other cognitive domains, involving memory function (Auditory Verbal Learning Test, AVLT; WMS-LM, immediate and delayed memory), language (Boston Naming Test, BNT), attention (Trail Making Test part A, TMT-A) and executive functioning (Trail Making Test part B, TMT-B). NC were only assessed by the MMSE, MoCA and WMS-LM, immediate and delayed memory.

### MRI Data Acquisition

We acquired both the structural and resting-state functional image data on a 3.0-Tesla GE Discover 750 MRI Scanner. Foam padding and earplugs were used to limit head motion and reduce scanner noise. T1-weighted images were acquired using a Sag FSPGR 3D ACC sequence with the following parameters: repetition time (RT) = 7.336 ms; echo time (TE) = 3.036 ms; inversion time (TI) = 450 ms; 196 sagittal slices; within plane FOV = 256 × 256 mm^2^; voxel size = 1.02 × 1.02 × 1.2 mm^3^; flip angle = 11°; and bandwidth = 244.141 Hz/pix. Resting-state functional MRI (rsfMRI) images were obtained with the following parameters: 205 time points; TR = 2,000 ms; TE = 30 ms; flip angle = 77°; number of slices = 38; slice thickness = 4 mm; spatial resolution = 3.75 × 3.75 × 4 mm^3^; and matrix = 64 × 64. DTI was obtained using the Ax DTI 30 sequence with the following parameters: 30 non-linear directions, *b*-value = 1,000 s/mm^2^, TR/TE = 6,000 ms/80.8 ms, 35 axial slices, slice thickness = 2 mm, FOV = 256 mm × 256 mm^2^, acquisition matrix = 128 × 128, and number of averages = 1. According to the scan protocol, all subjects were instructed to close their eyes and keep at rest calmly during the course of the scan.

### Imaging Pre-processing and VMHC Estimation

We pre-processed all data using the Data Processing Assistant and Resting-State FMRI (DPARSF, version 2.2; Chao-Gan and Yu-Feng, 2010,^1^) with Statistical Parametric Mapping 8 (SPM8^2^) on the MATLAB platform. First, we discarded the first 10 image volumes of rsfMRI scans due to the signal equilibrium and subject’s adaptation to the scanning noise. Then, we corrected the remaining 195 images for timing differences (37th slice as the reference slice) and head motion (24 parameters; Friston et al., 1996). Here, we discarded the image data with more than 2.5 mm maximum displacement in any of the x, y, or z directions or 2.5° of any angular motion (two EOAD patients excluded Supplementary Material, Part 6). Subsequently, based on rigid-body transformation, we coregistered the T1-weighted image to the mean rsfMRI image and spatially normalized these images to the Montreal Neurological Institute (MNI) space. A standardized image was subsequently re-sampled into 3 mm × 3 mm × 3 mm cubic voxels. To decrease spatial noise, we smoothed the rsfMRI images with a Gaussian kernel of 8 mm × 8 mm × 8 mm full width at half maximum (FWHM). We also performed a detrend and filter (0.01 Hz < *f* < 0.08 Hz) to discard the bias from the high-frequency physiological noise and the low-frequency drift. Finally, data were scrubbed to further reduce motion-related artifacts by using a frame-wise displacement (FD) threshold of 0.5, with which bad points were interpolated using the nearest neighbor (Yan et al., 2013). To remove residual effects of motion and other non-neuronal factors, we controlled for covariates including 24 head motion parameters and signals of WM and cerebrospinal fluid (CSF). Additionally, we considered the FD value of each subject as a covariate in a subsequent analysis procedure.

Regarding the VMHC analysis, we transformed pre-processed images by applying the rsfMRI image to a symmetric brain template, obtained by flipping the left or right hemispheres along the X-axis midline and averaging with the original image. Then, the T1 and rsfMRI images from each subject that had been normalized to the MNI space were co-registered nonlinearly to this group-specific symmetric template. Finally, we estimated the homotopic RSFC based on the Pearson correlation between every pair of symmetrical interhemispheric voxels’ time series. The resultant correlations constituted the VMHC map, followed by Fisher’s Z transformation. Notably, we used a unilateral brain mask to present results because the VMHC results were symmetric.

### Seed-Based Analysis

To reflect the all-around functional connectivity, seed-based analyses were complementarily performed. Five seed region of interests (ROIs) were selected to anchor the Default Mode Network (DMN), the left executive control network, the right executive control network, the language network, and the higher visual network (Lehmann et al., 2015). The average time series were extracted from 8 mm spheres around the peak intensity voxels. Then, a general analysis was performed to produce a subject-level intrinsic connectivity map with age, education and gender as covariates. The statistical analysis and results are listed in Supplementary Material, Part 1.

### Tract-Based Spatial Statistics (TBSS)

We analyzed DTI data following the standard procedure with PANDA version 1.3.0^3^ (Cui et al., 2013). Tract-based spatial statistics (TBSS) were used to calculate the diffusivity indices. First, we corrected eddy current distortions by registering the DW images to a b0 image with an affine transformation. Then, voxel-wise maps of FA, MD, AxD and RD were calculated with the DTIFIT program. Subsequently, a mean FA image was created by directly registering individual FA volumes to the FMRIB58 template (Smith et al., 2006) by non-linear registrations and thinned to represent the mean FA skeleton (FA > 0.25 overlaid with the mean FA image). Finally, the diffusion index data from individual subjects were projected onto this skeleton to create individual images.

### Gray Matter Volume Analysis

The T1-weighted images were pre-processed using the Computational Anatomy Toolbox (CAT12^4^). First, the T1 image was spatially registered to the tissue probability maps (TPM) and then segmented into gray matter (GM), WM and CSF. ICBM152 space was used to perform the affine registration to the stereotactic MNI space. Then, high-dimensional DARTEL normalization and nonlinear modulation using the Jacobian determinants derived from the normalization process were performed. Subsequently, we smoothed the GM image with a Gaussian kernel of 8 mm × 8 mm × 8 mm to reduce potential inaccuracies during the normalization step. Moreover, we extracted the GM volume (GMV) in every ROI based on the Hammers atlas provided by CAT12 and used these data in subsequent analyses.

### Statistical Analyses

We analyzed the demographic data using the Chi-squared test for categorical data and *t*-test for continuous data (SPSS version 19.0).

Then, we examined the VMHC differences between EOAD and LOAD in a voxel-wise manner based on REST software^5^). In detail, we performed a two-sample *t*-test between the patient and control groups by setting the statistical threshold at *P* < 0.001 and cluster size >10 voxels (uncorrected). Moreover, to evaluate the impact of WM lesion, we additionally performed the analysis by adding WM load as a covariate. For detail, Supplementary Material, Part 5.

Regarding the DTI results, voxel-wise group comparisons were carried out using non-parametric, two-sample *t*-tests (randomize, Smith et al., 2006) in the patient vs. its corresponding control group (corrected by threshold-free cluster enhancement *p* < 0.05). We used the mean FA skeleton as a mask with a threshold of 0.25 and set the number of permutations at 5,000 (Smith and Nichols, 2009).

Regarding GMV results, voxel-wise group comparisons were performed using the two-sample *t*-test between the EOAD patients, LOAD patients and age-matched controls with age, gender, and education as the covariates. Moreover, a significant threshold (*P* < 0.005, cluster size >500) was applied after correction for multiple comparisons using a false discovery rate (FDR) procedure. Moreover, ROI-based group comparisons were performed in patients vs. NC, within the young and old groups (mainly focusing on HP and medial temporal lobe (MTL)).

We defined brain regions showing significant between-group differences as ROIs and extracted the values within them. Then, we conducted Pearson correlation analyses between the interhemispheric RSFC, diffusion indices, GMV and behavioral data. Moreover, regression analysis with neuropsychological tests as dependent variables and age of onset, gender, education and neuroimaging data as independent variables were performed to define whether neuroimaging variables predicted cognitive functions and whether the diagnostic group impacts these correlations.

## Results

### Demographics

The EOAD and LOAD patients matched well with their respective controls for age, gender, education level, GDS and FD value (*P* > 0.05, Table 1). Regarding the neuropsychological profiles, both EOAD and LOAD patients performed worse than their respective controls in MMSE, MoCA, WMS-LM and AVLT scores (*P* < 0.05, Table 2).

**Table 2:** Neuropsychological test performance of early-onset and late-onset Alzheimer’s disease (AD) patients.

Variables	EOAD patients	Young controls	EOAD vs. Young controls (*p* value)	LOAD patients	Old controls	LOAD vs. Old controls (*p* value)	EOAD vs. LOAD (*p* value)
Memory function							
WMS-LM immediate	3.48 ± 3.11	10.00 ± 3.61	<0.001*	3.48 ± 2.17	8.00 ± 4.30	<0.001*	0.94
WMS-LM delay	0.76 ± 1.58	8.14 ± 3.13	<0.001*	0.30 ± 0.72	6.43 ± 3.78	<0.001*	0.18
AVLT sum of trials 1–5	18.14 ± 11.76			18.30 ± 6.24			0.96
Attention							
TMT-A	100.16 ± 43.51			101.04 ± 36.58			0.94
Decision-making function							
TMT-B	188.21 ± 106.19			233.06 ± 87.74			0.18
Language							
BNT	11.38 ± 8.26			13.29 ± 4.84			0.37

Values are expressed as the mean ± standard deviation or number of participants. *p < 0.05, significant difference. EOAD, early-onset Alzheimer’s disease; LOAD, late-onset Alzheimer’s disease; WMS-LM, Wechsler Memory Scale-Revised Logical Memory II; AVLT, Auditory Verbal Learning Test; TMT, Trail-Making Test; BNT, Boston naming test.

### Interhemispheric Functional Connectivity Differences

**Figure 1:**
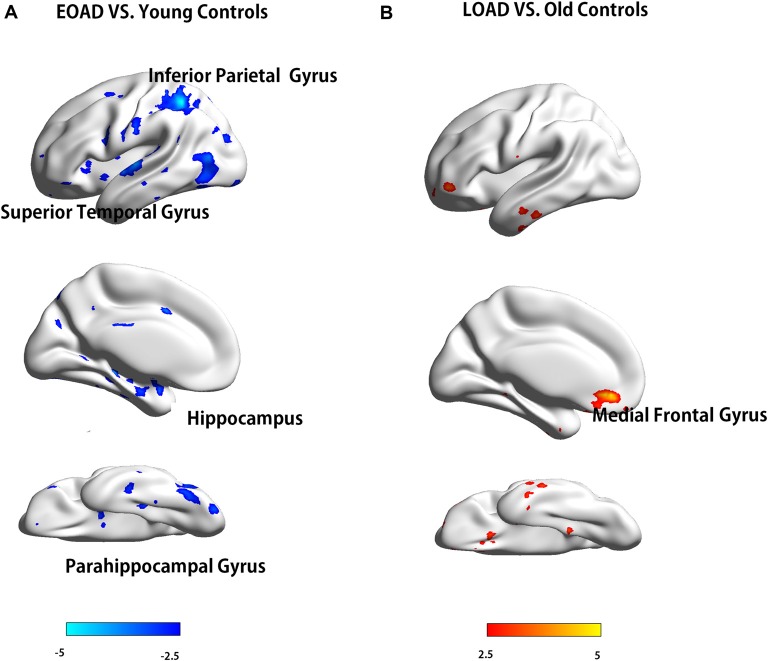
Difference in VMHC between patients and controls (EOAD patients vs. young controls; LOAD patients vs. old controls). Comparison of interhemispheric RSFC between patients and controls. **(A)** The EOAD patients showed significantly decreased interhemispheric RSFC in the HP, PHG, STG and IPC. **(B)** The LOAD patients had increased VMHC in the MFG (*P* < 0.001, cluster size >10 voxels, uncorrected). Note: only the left side of the image was displayed due to the symmetric template used in the analysis. VMHC, voxel-mirrored homotopic connectivity; EOAD, early-onset Alzheimer’s disease; LOAD, late-onset Alzheimer’s disease; RSFC, resting-state functional connectivity; HP, hippocampus; PHG, parahippocampal gyrus; STG, superior temporal gyrus; IPC, inferior parietal cortex; MFG, medial frontal gyrus.

The EOAD patients showed lower interhemispheric RSFC in the hippocampus (HP), parahippocampal gyrus (PHG), superior temporal gyrus (STG) and inferior parietal cortex (IPC) than the young controls (Figure 1). In contrast, the LOAD patients showed greater interhemispheric RSFC in the medial frontal gyrus (MFG) than the old controls (Table 3 and Figure 1).

**Table 3:** Brain regions with different VMHC between the two groups.

	Brain regions	Cluster size	Coordinates(MNI)			Peak intensity
			*x*	*y*	*z*	
Young controls > EOAD	Parahippocampal gyrus_L	14	-18	-3	-27	4.93
	Hippocampus_L	10	-33	-33	-9	4.60
	Temporal_Sup_L	11	-57	-15	3	4.44
	Parietal_Inf_L	67	-33	-51	51	5.57
LOAD > Old controls	Frontal_Med_Orb_L	19	-6	39	-9	5.84

### Interhemispheric Structural Connectivity Differences

**Figure 2:**
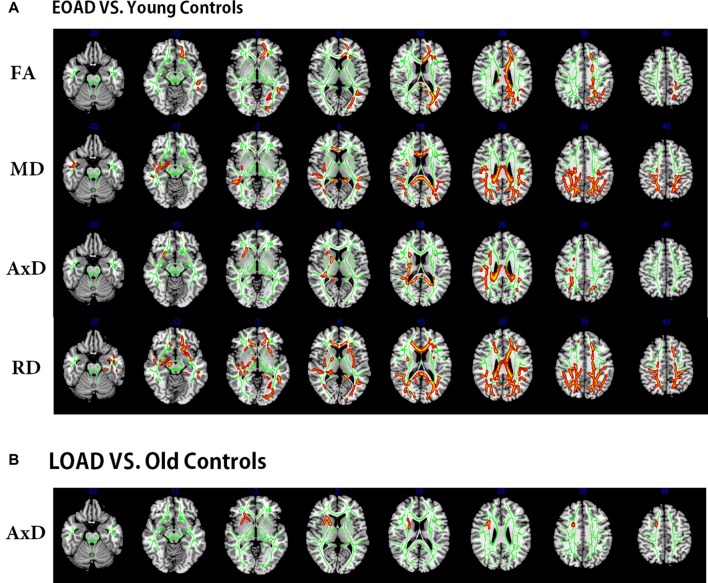
Tract-based spatial statistics (TBSS) results of the diffusion indices between the patients and controls (EOAD patients vs. young; LOAD patients vs. old controls). Green represents the mean white matter (WM) skeleton of all subjects. **(A)** Red-yellow voxels represent the WM regions with reduced FA (first row), increased MD (second row), increased AxD (third row), and increased RD (fourth row) in the EOAD patients compared with the young controls (*p* < 0.05, corrected). **(B)** Red-yellow voxels represent the WM regions with increased AxD in the LOAD patients compared with the old controls (*p* < 0.05, corrected). EOAD, early-onset Alzheimer’s disease; LOAD, late-onset Alzheimer’s disease; FA, fractional anisotropy; MD, mean diffusivity; AxD, axial diffusivity; RD, radial diffusivity.

Compared to the young controls, the EOAD patients showed reduced FA in widespread WM regions (posterior thalamic radiation, genu of CC and anterior corona radiata) and increased MD (in posterior parts of the brain, including the genu of CC, CC splenium and parietooccipital regions). Additionally, the EOAD patients showed increased AxD (in the body of CC, bilateral superior corona radiata, CC splenium and parietooccipital regions) and increased RD (in the posterior corona radiata, anterior corona radiata, right fornix, right cerebral peduncle, right external capsule and inferior temporal gyrus, Figure 2).

The LOAD patients only showed increased AxD in the right anterior corona radiata (Figure 2) when compared to AxD in the old controls. No significant difference was observed between the LOAD patients and old controls in FA, MD and RD indices (overlap map of VMHC and DTI in Supplementary Material, Part 2).

### Gray Matter Volume Difference

The EOAD patients showed decreased GMV in the PHG and IPC, while the LOAD patients showed decreased GMV in the PHG, compared to their respective controls. Moreover, the ROI-based comparison showed that the LOAD patients had a smaller HP and MTL volume than the EOAD patients (Supplementary Material, Part 3).

### Correlation of Interhemispheric Connectivity With Behavioral Data

**Figure 3:**
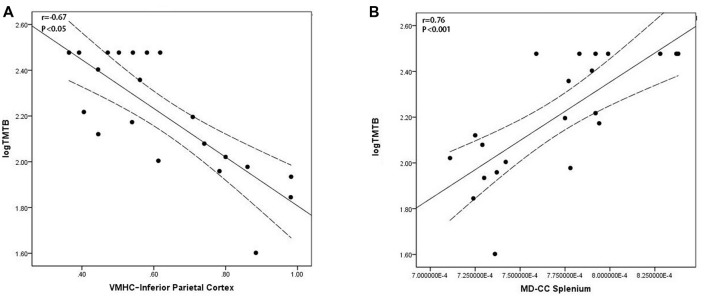
Scatter plot diagram of the correlation between executive performance and VMHC/diffusion indices in the EOAD patients. Scatter plot diagram displaying the 95% confidence band of the best-fit line. **(A)** The decreased VMHC of the IPC was negatively correlated with executive function (*r* = −0.67, *P* < 0.05). **(B)** The increased MD of the CC splenium was positively correlated with executive function (*r* = 0.76, *P* < 0.001). VMHC, voxel-mirrored homotopic connectivity; EOAD, early-onset Alzheimer’s disease; IPC, inferior parietal cortex; MD, mean diffusivity; CC, corpus callosum.

In the EOAD group, the VMHC of the IPC was negatively associated with the TMT-B scores (*r* = −0.67, *P* < 0.05, Figure 3A) and MD of the CC splenium positively associated with the TMT-B scores (*r* = 0.76, *P* < 0.001, Figure 3B). In the LOAD group, GMV in the HP positively correlated with the AVLT scores (*p* = 0.005, *r* = 0.56; Supplementary Material, Part 3). Similar results were also found in the regression analysis (Supplementary Material, Part 4).

## Discussion

Building on multi-modal MRI methods, we found distinct impairment patterns of interhemispheric functional and structural connectivity in the EOAD and LOAD patients. Our results showed that: (1) the EOAD patients had widespread decreased VMHC relative to the LOAD patients; (2) meanwhile, the EOAD patients showed extensive WM deficits, especially in the posterior regions. In contrast, only the medial frontal regions showed changes in diffusion indices in the LOAD patients compared with old controls; and (3) decreased communication between the bilateral IPC and corresponding deficits in interhemispheric structural pathways contributed to the executive function impairment within the EOAD patients.

In this study, we observed decreased interhemispheric RSFC in the HP and PHG in the EOAD patients. These structures are in charge of information encoding and convergence (Eichenbaum, 2000) and are tightly associated with declarative memory (Burwell et al., 1995; Suzuki, 1996). Previous studies have reported that these regions are susceptible to AD-related pathophysiological changes (Braak and Braak, 1991; Van Hoesen et al., 2000), which eventually cause neuronal damage and cell death (Yan et al., 1996; Hardy and Selkoe, 2002). Morphological studies may support these findings by indicating cortical atrophy in the medial temporal and parahippocampal regions in EOAD patients (Ishii et al., 2005; Möller et al., 2013; Caso et al., 2015). Functionally, our results extended previous rsfMRI studies that documented impaired functional connectivity in the HP and PHG in EOAD (Adriaanse et al., 2014; Gour et al., 2014) and further found interhemispheric functional connectivity impairments. However, we would note that Lehmann et al. (2015) once found the opposite results that EOAD displayed preserved DMN connectivity. Our seed-based functional analysis also found similar results, which showed relatively preserved DMN connectivity but more non-DMN connectivity impairments in EOAD (for details see Supplementary Material, Part 3). We inferred that the different methods might explain this discrepancy because VMHC reflects interhemispheric connectivity, while seed-based connectivity analysis focuses more on the intra-network function. On the other hand, our results showed that the STG had decreased interhemispheric functional connectivity in the EOAD patients. Although the STG is not typically a primary target site in AD, the STG possesses polysensory information interactions with the PHG (Suzuki and Amaral, 1994) and is involved in both semantic and syntactic processing. Prior studies also reported that EOAD patients had hypoperfusion and hypometabolism in the lateral temporal cortex (Ichimiya et al., 1994; Kemp et al., 2003; Frisoni et al., 2005; Shiino et al., 2008; Migliaccio et al., 2015). Furthermore, evidence from [11C] PiB-PET also suggested that decreased VMHC in the STG may derive from amyloid deposition (Cho et al., 2013). Moreover, decreased ceruloplasmin levels, which reflects decreased cellular metabolic processes and protective ability against oxidative damage, in the STG in AD patients may be another reason (Connor et al., 1993). In summary, our results suggested interhemispheric functional disruption in the EOAD patients involving the cortical-hippocampal system.

The EOAD patients also had decreased functional connectivity between the bilateral IPC. The IPC is regarded as a component of the executive frontoparietal network (Seeley et al., 2007) and showed functional and structural impairments in EOAD patients. Functionally, Gour et al. (2014) reported decreased functional connectivity in EOAD patients in the IPC. This was also proven by our seed-based network analysis and suggested its vulnerability to functional impairments in both interhemispheric and intra-network connectivity Supplementary Material, Part 1). Structurally, prior studies demonstrated that patients with EOAD had more severe atrophy and hypometabolism than LOAD patients in parietal regions (Sakamoto et al., 2002; Ishii et al., 2005; Kim et al., 2005; Möller et al., 2013; Verclytte et al., 2016). Specifically, Ossenkoppele et al. (2012) found both an increased amyloid burden and metabolic dysfunction in parietal regions in EOAD; further analysis found metabolic impairment in the parietal cortex was related to executive function and attention (Ossenkoppele et al., 2012). This evidence is consistent with our regression analysis results showing that IPC VMHC and DTI in the parietooccipital region independently contribute to executive function in EOAD. Thus, we speculated that information integration processes between the bilateral IPC might contribute to executive function.

Considering the strong link between functional and structural connectivity, we proposed that WM integrity abnormalities may underlie the pathological mechanisms of VMHC deficits in EOAD patients. In particular, we found widespread WM alterations in the EOAD patients, especially in the CC, corona radiata, thalamic radiation and posterior brain regions. Similarly, previous studies reported WM alterations in EOAD patients, mainly involving the CC and dorsal temporoparietal regions (Canu et al., 2012; Caso et al., 2015; Daianu et al., 2016). Moreover, Caso et al. (2015) reported WM damage in the medial temporal and parahippocampal regions in EOAD patients. Anatomically, these WM tracts work as the basic structure for information exchange between hemispheres (Wakana et al., 2004; Silk et al., 2009). More specifically, the CC splenium connects the temporal, parietal, and occipital regions (de Lacoste et al., 1985), while the fornix works as the major afferent and output tract of the HP (Aggleton and Brown, 1999). Thus, we hypothesized that WM impairments in the posterior regions contributed to VMHC reduction in the temporal and parietal regions in the EOAD patients. Notably, we also found an association between the CC splenium microstructural breakdown and executive function in the EOAD patients. The high consistency across different imaging modalities mutually provided conclusive support for our findings in the EOAD patients.

Regarding the LOAD patients, we found increased interhemispheric RSFC with impaired WM in the orbital part of the MFG. Previous functional studies also found increased connectivity in the frontal regions and assumed it to be a compensatory effect in LOAD patients (Agosta et al., 2012; Adriaanse et al., 2014; Gour et al., 2014). Additionally, Gour et al. (2014) reported increased functional connectivity in frontal regions and its correlation with executive function in LOAD patients. This is partially in line with our network analysis, which found increased connectivity in the prefrontal region in the LOAD patients. Our VMHC results validated these findings by demonstrating increased interhemispheric functional connections in frontal regions and its corresponding WM impairments. Moreover, we found greater GMV loss in the LOAD patients, especially in the medial temporal region, which was significantly correlated with memory. Regression analysis showed that GMV in the medial temporal region independently contributed to memory in the LOAD patients. Thus, we proposed a possible mechanism underlying LOAD: GMV loss, rather than interhemispheric connectivity, was more related to cognitive decline in the LOAD patients. Although the LOAD patients had severe GM loss, the relatively well-preserved WM structure provided the structural base for interhemispheric functional connectivity compensation.

Unexpectedly, we found no interhemispheric functional connectivity decreases in the LOAD patients. Our observation is somewhat different from previous studies demonstrating functional impairments in memory-related regions in LOAD patients (Hodges, 2006; de Waal et al., 2012; Adriaanse et al., 2014; Gour et al., 2014). Some reasons may account for this inconsistency. First, GM atrophy usually precedes WM degeneration in AD (Reid and Evans, 2013). The current study mainly focused on AD patients at an early clinical stage, which may be associated with less WM impairments. At this stage, the interhemispheric cooperation may still mostly preserve function in the LOAD patients. In addition, different cultural customs between Eastern and Western elderly should be noted. In contrast to most previous studies, our study was conducted in Chinese elderly. For example, eating food with chopsticks may significantly improve the interhemispheric communication in LOAD patients, who have relatively milder forms of AD pathologies than EOAD patients have. The work of Demirakca investigated the impact of life training on brain plasticity and found strengthened interhemispheric functional connectivity (Demirakca et al., 2016).

There were some limitations to this study. First, the small sample size decreased the statistical power. Thus, the findings should be replicated in a larger sample in the future. Second, the natural human brain is not entirely symmetrical. However, we co-registered rsfMRI data to a symmetrical structural T1 template and measured the functional correlations between mirrored regions. Although our interhemispheric structural connectivity results mostly validated the functional results, this potential bias cannot be neglected in future studies. Third, our study mainly focused on subjects in the early stages of AD with a relatively high MMSE score, which may not be representative of the whole spectrum of AD severity. In future studies, we should include more AD patients at different stages. Finally, the absence of PET amyloid data is another limitation considering its importance in the AD diagnosis.

## Conclusion

In summary, we observed different impairment patterns of interhemispheric connectivity in the EOAD and LOAD patients based on multi-modal imaging methods. The EOAD patients showed widespread interhemispheric impairments of the MTL, STG, and IPC. However, we found a compensatory effect of frontal interhemispheric communication in the LOAD patients. Correspondingly, the WM alterations showed a large overlap with the functional changes, suggesting its role as the structural base of bi-hemispheric functional changes.

## Author Contributions

K-CL designed the study and wrote the first draft of the manuscript. XL analyzed the MRI data and wrote the protocol. Q-ZZ and J-JX collected the clinical and MRI data. JZ diagnosed and collected the patients. X-JX, P-YH, Z-JS and M-MZ assisted with the study design and interpretation of findings. All authors have contributed to and approved the final manuscript.

## Conflict of Interest Statement

The authors declare that the research was conducted in the absence of any commercial or financial relationships that could be construed as a potential conflict of interest.

## Footnotes

^1^http://rfmri.org/DPARSF

^2^www.fil.ion.ucl.ac.uk/spm/

^3^www.nitrc.org/projects/panda/

^4^http://dbm.neuro.uni-jena.de/cat12/

^5^www.restfmri.net
