# Supplementary material for: Distinct Patterns of Interhemispheric Connectivity in Patients With Early- and Late-Onset Alzheimer’s Disease
Source: Front Aging Neurosci. 2018 Sep 6;10:261. doi: 10.3389/fnagi.2018.00261 (PMC6136638; doi:10.3389/fnagi.2018.00261)
Supplement: Supplementary file 1 [file Data_Sheet_1.pdf]

*Supplementary Material*

# Distinct Patterns of Interhemispheric Connectivity in Patients with Early- and Late-Onset Alzheimer's Disease

Kai-Cheng Li[1†], Xiao Luo[1†], Qing-Ze Zeng[1] , Xiao-Jun Xu[1], Pei-Yu Huang[1], Zhu-Jing Shen[1], Jing-Jing Xu [1], Jiong Zhou[2], Min-Ming Zhang*[1]

**Corresponding to:**

*Prof. Minming Zhang, MD, Ph.D.; Department of Radiology, The 2nd Affiliated Hospital of Zhejiang University, School of Medicine, No.88 Jie-fang Road, Shang-cheng District, Hangzhou, China, 310009; Phone: 86-0571-87315255; Fax: 86-0571-87315255;

**Email address**: zhangminming@zju.edu.cn

# Part 1

VMHC can effectively reflect the process of exchange and the integration of information between the cerebral hemispheres, but it may not be informative in terms of brain circuits. However, seed-based analysis can effectively reflect the functional network connectivity and has been used to explore network changes in EOAD. The work of Lehmann et al. reported relatively preserved DMN connectivity but more non-DMN impairments in EOAD patients [1]. However, another study found similar reductions in DMN connectivity in EOAD and LOAD patients compared with controls [2]. To verify the network changes in the two subtypes of AD, seed-based analysis was also performed as a complementary analysis.

Five seed ROIs were selected to anchor the DMN (right precuneus, MNI 2 −68 36), the left executive control network (left angular gyrus, MNI −38 −68 48), the right executive control network (right supramarginal gyrus, MNI 52 −46 48), the language network (left middle temporal gyrus, MNI −54 −56 22), and the higher visual network (right middle occipital gyrus, MNI 36 −88 0) [1]. The average time series were extracted from eight millimetre spheres around the peak intensity voxels. Then, a general analysis was performed to produce a subject-level intrinsic connectivity map with age, education and gender as covariates. Furthermore, we performed voxel-wise two-sample t-tests between patients and the respective control group to examine the functional connectivity difference, setting the threshold at the height level $p<0.05$ and cluster level at $p<0.05$ (Gaussian random field correction).

The results showed that the EOAD patients had decreased functional connectivity in a higher visual network and language network when compared to the young controls. No significant difference was found in the DMN between the EOAD patients and the young controls. The LOAD patients showed increased functional connectivity in both the DMN and visual network when compared to the old controls. No significant connectivity difference in the executive network was found in either subtype group (Table 1, Figure 1).

Such a result is partially in line with previous study which reported relatively preserved DMN connectivity but more non-DMN connectivity impairments in EOAD [1]. We inferred that the different method might interpret this discrepancy since VMHC reflects inter-hemispheric connectivity while seed-based connectivity analysis focuses more on the intra-network function. As for LOAD part, it can provide the effective evidence for relatively intact functional connectivity in LOAD.

**Table 1. Network difference between patients and controls by using seed-based connectivity analysis**

| Network | Group | Brain Regions | Cluter-size | Coordinates(MNI) | | | Peak intensity |
|---|---|---|---|---|---|---|---|
| | | | | x | y | z | |
| DMN | EOAD VS. young controls | no | | | | | |
| | LOAD VS. old controls | Temporal_Mid_L | 2740 | -36 | 3 | -48 | 4.3137 |
| | | Frontal_Mid_R | 3082 | 36 | 33 | 4 | 3.93 |
| Language Network | EOAD VS. young controls | Temporal_Mid_R | 1896 | -9 | 24 | -15 | -3.9519 |
| | LOAD VS. old controls | no | | | | | |
| Higher Visual Network | EOAD VS. young controls | Inferior Parietal Lobule | 15039 | -45 | -27 | 33 | -6.2514 |
| | LOAD VS. old controls | Frontal_Mid_R | 1146 | 39 | 9 | 24 | 4.5231 |

EOAD: early-onset Alzheimer's disease; LOAD: late-onset Alzheimer's disease; DMN, default mode network

**Figure 1. Network difference between patients and controls by using seed-based connectivity analysis**

The EOAD patients showed decreased functional connectivity in higher visual network and language network when compared to young controls. The LOAD patients showed increased functional connectivity in both DMN and higher visual network when compared to old controls. No significant connectivity difference was found in executive network in either subtype group.

EOAD: early-onset Alzheimer's disease; LOAD: late-onset Alzheimer's disease; DMN, default mode network

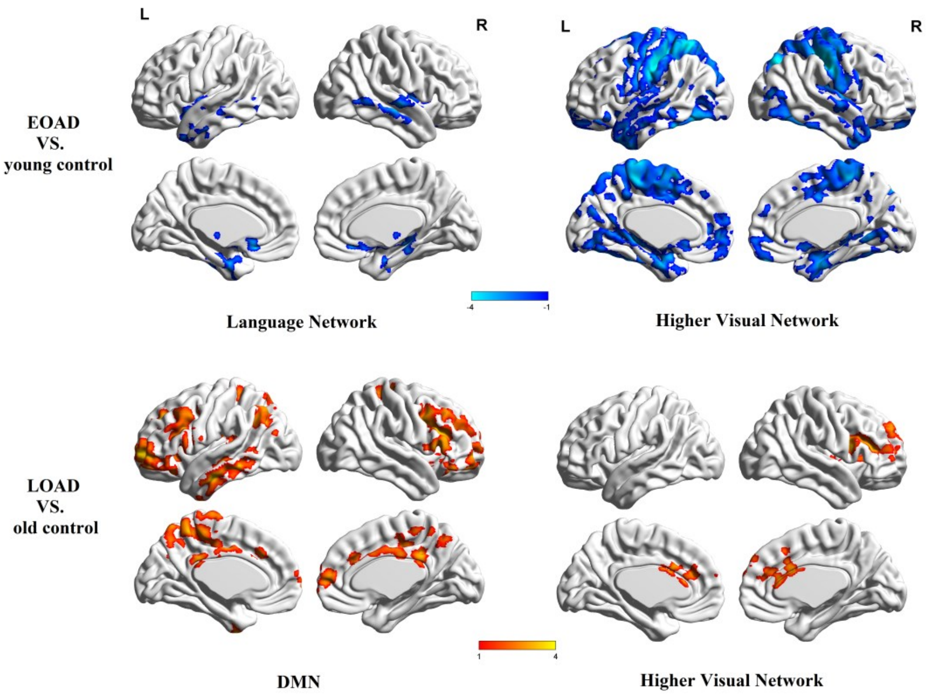

# Part 2

**Figure 1. Overlap map of VMHC and DTI (EOAD patients *vs.* young controls; LOAD patients *vs.* old controls)**

A symmetric brain template was used to showed the overlap of VMHC and DTI in the whole brain. The EOAD patients showed decreased VMHC in hippocampus (HP), parahippocampal gyrus (PHG), superior temporal gyrus (STG), and inferior parietal cortex (IPC) which is almost overlapped with white matter impairments. The LOAD patients showed increased inter-hemispheric RSFC while impaired white matter in the orbital part of medial frontal gyrus.

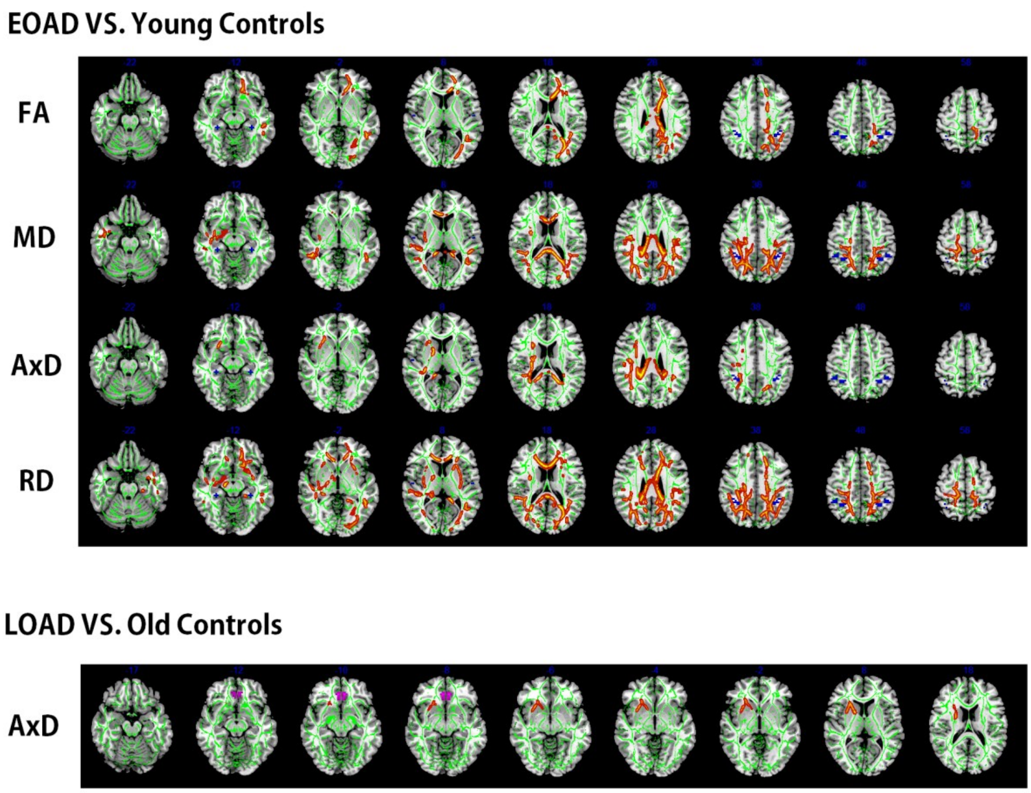

**Difference of white matter between patients and controls (EOAD patients *vs.* young controls; LOAD patients *vs.* old controls)**

**Table 1. White matter difference between EOAD and young controls**

Compared to the young controls, the EOAD patients showed impaired diffusion indices in widespread white matter regions, especially in the posterior region. For detail, see the following table and results part in the manuscript.

| Brain Regions | Coordinates(MNI) | | | Cluster Size |
|---|---|---|---|---|
| | x | y | z | |
| **FA** | | | | |
| Posterior Thalamic Radiation_L | 116 | 59 | 86 | 5515 |
| Genu of corpus Callosum | 102 | 152 | 88 | 3212 |
| Anterior Corona Radiata_L | 115 | 158 | 73 | 172 |
| Anterior Corona Radiata_L | 110 | 155 | 65 | 51 |
| Anterior Corona Radiata_L | 115 | 151 | 88 | 30 |
| Anterior Corona Radiata_L | 114 | 151 | 62 | 19 |
| **MD** | | | | |
| parietooccipital region | 121 | 63 | 102 | 17648 |
| Genu of corpus Callosum | 84 | 156 | 73 | 492 |
| Genu of corpus Callosum | 96 | 154 | 82 | 120 |
| posterior region of Brain | 110 | 89 | 78 | 21 |
| Splenium of Corpus Callosum | 103 | 83 | 99 | 14 |
| **AxD** | | | | |
| Body of Corpus Callosum | 79 | 96 | 97 | 3351 |
| Superior Corona Radiata_R | 63 | 127 | 99 | 1004 |
| Splenium of Corpus Callosum | 106 | 82 | 94 | 823 |
| parietooccipital region | 121 | 64 | 103 | 216 |
| Superior Corona Radiata_L | 126 | 86 | 97 | 105 |
| **RD** | | | | |
| Posterior corona radiata | 111 | 70 | 97 | 25058 |
| Fornix(cres) | 71 | 90 | 79 | 946 |
| Cerebral Pedunle_R | 77 | 109 | 56 | 465 |
| Anterior Corona Radiata | 64 | 148 | 69 | 94 |
| External Capsule_R | 58 | 135 | 75 | 18 |
| Inferior frontal gyrus | 37 | 82 | 57 | 18 |

**Table2: White matter difference between LOAD and old controls**

The LOAD patients only showed increased AxD in right anterior corona radiata when compared to old controls.

| Brain Regions | Coordinates(MNI) | | | Cluster Size |
|---|---|---|---|---|
| | x | y | z | |
| **AxD** | | | | |
| External Capsule_R/Anterior Corona Radiata | 60 | 142 | 74 | 1499 |

# Part 3

**Difference of GMV between patients and controls (EOAD patients vs. young controls; LOAD patients vs. old controls)**

Voxel-wise comparison of GMV between patients and controls. A). The EOAD patients showed significantly decreased GMV in PHG and IPC. B). The LOAD patients showed decreased GMV in PHG. (FDR, P<0.005, cluster size>500 voxels)

GMV: grey matter volume; EOAD: early-onset Alzheimer's disease; LOAD: late-onset Alzheimer's disease; PHG: parahippocampal gyrus; IPC: inferior parietal cortex; MTL: medial temporal lobe

**Table 1. Voxel-wise GMV Comparison between two groups**

| | Brain Regions | Cluter-size | Coordinates(MNI) | | | Peak intensity |
|---|---|---|---|---|---|---|
| | | | x | y | z | |
| Young controls>EOAD | Parahippocampa Gyrus | 2602 | 3 | 0 | 3 | -7.2593 |
| | Parietal_Inf_L | 727 | -27 | -69 | 39 | -5.9058 |
| Old controls>LOAD | parahippocampa gyrus_R | 535 | 18 | 3 | -24 | -6.1651 |

GMV: grey matter volume
EOAD: early-onset Alzheimer's disease; LOAD: late-onset Alzheimer's disease;
FDR P<0.005 cluster size>500

**Figure 1. Grey Matter Difference between Patients and Controls**

**A). EOAD *VS.* young controls**

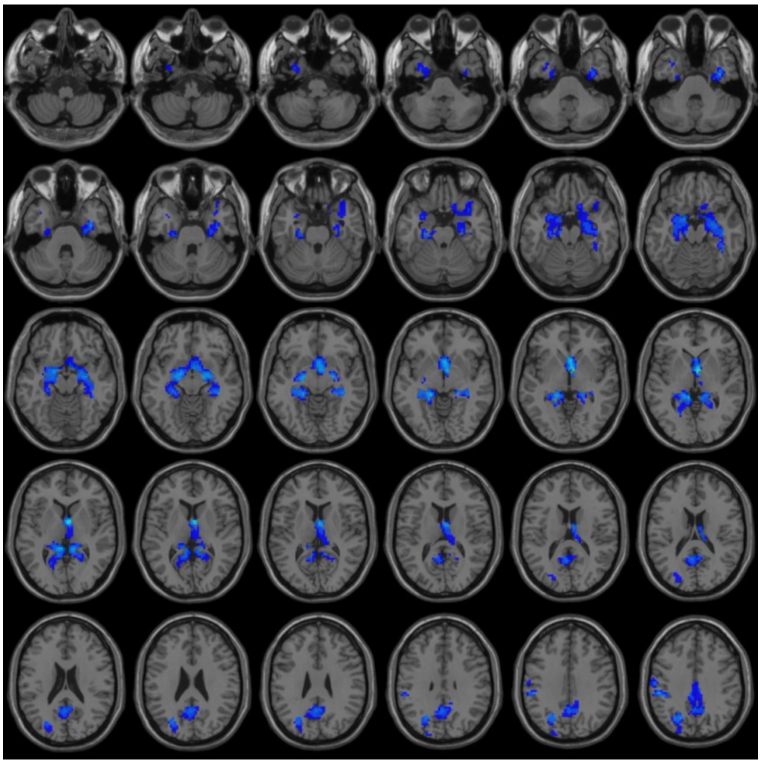

**B) LOAD *VS.* old controls**

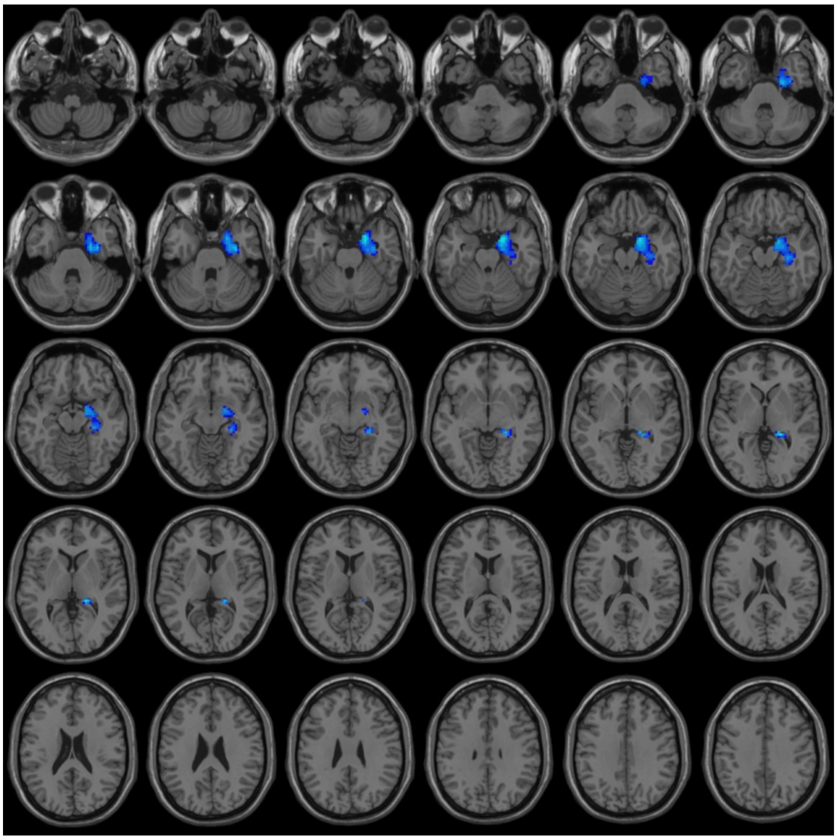

**Table 2. ROI-based GMV Comprison between Patients and Controls**

ROI-based comparison of GMV in the HP and MTL between patients and controls. The EOAD patients showed significantly decreased GMV in the HP and MTL when compared to young controls;. The LOAD patients showed decreased GMV in the HP and MTL when compared with old controls; The LOAD patients showed decreased GMV in HP and MTL when compared with the EOAD patients.

In the LOAD group, GMV in the HP was positively correlated with AVLT (p<0.001, r=0.72)

GMV: grey matter volume; EOAD: early-onset Alzheimer’s disease; LOAD: late-onset Alzheimer’s disease; HP: hippocampus; MTL: medial temporal lobe; AVLT: Auditory Verbal Learning Test

| Variables | EOAD patients (n = 22) | Young controls (n = 21) | EOAD VS Young controls (p value) | LOAD patients (n=27) | Old controls (n=17) | LOAD VS Old controls (p value) | EOAD VS LOAD (p value) |
|---|---|---|---|---|---|---|---|
| right HP | 1.89±0.40 | 2.31±0.21 | < 0.001* | 1.61±0.29 | 2.04±0.23 | < 0.001* | 0.008* |
| left HP | 1.77±0.40 | 2.20±0.24 | < 0.001* | 1.55±0.26 | 1.94±0.16 | < 0.001* | 0.024* |
| right anterior medial temporal lobe | 4.90±0.96 | 5.63±0.71 | 0.007* | 4.35±0.91 | 5.19±0.64 | 0.002* | 0.048* |
| left anterior medial temporal lobe | 4.69±0.88 | 5.58±0.76 | 0.001* | 4.19±0.84 | 5.19±0.79 | < 0.001* | 0.048* |

Data are presented as means ± standard deviations.
EOAD: early-onset Alzheimer's disease; LOAD: late-onset Alzheimer's disease;
*p < 0.05, significant difference

**Figure 2. Correlation between GMV of HP and AVLT (p<0.001, r=0.72)**

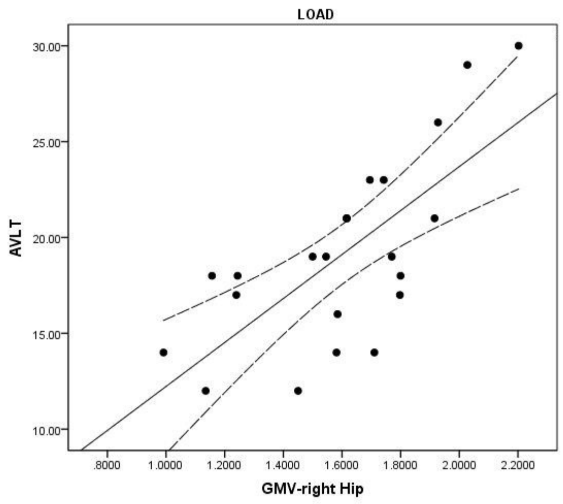


# Part 4

To explore whether neuroimaging variables predict cognitive functions, we performed the multivariate model analysis within EOAD patients, LOAD patients and the whole AD cases, respectively. The dependent variable included Auditory Verbal Learning Test (AVLT), Boston Naming Test (BNT), Trail Making Test part B (TMT-B), Trail Making Test part A (TMT-A) which reflects memory, language, executive function and attention, respectively. Z scores were calculated and used here.

Within the EOAD cases, independent variables included ROI-based VMHC (L-hippocampus (HP); L-parahippocampus gyrus (PHG); L-inferior parietal cortex (IPC); L-superior temporal gyrus (STG)); ROI-based GM volume (HP, IPC); ROI-based MD (parietooccipital region), age of onset, gender, and education. ROIs were chosed from the two sample t-test in GMV, VMHC and DTI between EOAD and young controls. To reduce the colinearity, we selected the MD as the diffusion index during the regression analysis. Results showed that white matter in the parietooccipital region and functional connectivity of IPC are independent contributing factors in executive function, since a significant regression model was found in MD, VMHC of IPC and TMTB (P<0.05, R2 =0.43, β=0.48 for and -0.46, respectively). This result is in line with our correlation finding and suggested that the functional and structural connectivity in IPC are important in executive function.

Within the LOAD cases, independent variables included ROI-based VMHC (L-medial orbital frontal gyrus); ROI-based GM volume (HP, medial temporal lobe (MTL)); ROI-based AxD (anterior corona radiata), age of onset, gender, and education. GMV ROIs here were chosed according to the Hammers atlas map provided by CAT12. Results showed that GMV in HP is an independent contributing factor in memory, since a significant regression model was found in GMV of HP and AVLT (P<0.05, R2 =0.45, β=69). This is line with our hypothesis: GMV loss may be the most prominent change in early LOAD patients while white matter tracts keep relatively intact. And such a GMV loss may account for the memory loss in early LOAD patients..

Within the whole AD cases, independent variables included ROI-based VMHC (L-HP; L-PHG; L-IPC; L-STG; L-medial orbital frontal gyrus); ROI-based GM volume (HP, MTL); ROI-based MD

(parietooccipital region) and, age of onset, gender, and education. GMV ROIs here were chosed according to the Hammers atlas map provided by CAT12. Results showed that GMV of HP is an independent contributing factor in memory, since a significant regression model was found between it and AVLT ($P<0.05$, R2 =0.20, β=0.47). Moreover, GMV of MTL is also a important factor in language, since a significant regression model was found between GMV of MTL and BNT ($P<0.05$, R2 =0.36, β=0.55).

Conclusively, Structural and functional connectivity work together to keep normal cognition. GM is the basic of neuron activity while white matter connectivity works as the base of functional connectivity. Within the whole AD groups, GM seems to be the most important factor in cognition. This is plausible since GM atroph is the well-established feature of AD. Moreover, multimode analysis in AD subtypes by combined with white matter and inter-hemispheric connectivity analysis can further reflect seperate underlying mechanism.

# Part 5

To evaluate the impact of white matter (WM) lesion, we calculated the WM load by using Lesion Segmentation Toolbox. Firstly, a two-sample T-test was performed to compare WM load between patients and controls. The EOAD patients have more WM load than younger controls while LOAD patients have no significant difference in WM load compared to old controls (Table 1). This result strongly supported our DTI results that EOAD patients feature more serious WM impairments than corresponding controls. Besides, we found more WM load in LOAD than EOAD patients. These results could be interpreted by the normal aging process because more WMH burden existed in old controls relative to young controls (Table 1).

According to review's comment, we also added WMH burden as the covariate during functional analysis. Results remained largely unchanged ( Table 2, Figure 1).

**Table 1. White Matter Lesion Difference**

| | EOAD | EONC | EOAD Vs. Young Controls P-value | LOAD | LONC | LOAD Vs. Old Controls P-value | EOAD Vs.LOAD P-value | Young Controls Vs. Old Controls P-value |
|---|---|---|---|---|---|---|---|---|
| white matter lesion | 18.62±17.66 | 7.73±8.81 | 0.015* | 28.78±15.06 | 21.00±13.58 | 0.091 | 0.035* | 0.002* |

*p < 0.05, significant difference between two groups

**Table 2. Different brain regions of VMHC between two groups (add WM load as the covariate)**

| | Brain Regions | Cluter-size | Coordinates(MNI) x | y | z | Peak intensity |
|---|---|---|---|---|---|---|
| Younger control>EOAD | parahippocampa gyrus_L | 15 | -18 | -3 | -27 | -5.6895 |
| | Hippocampus_L | 13 | -33 | -33 | -9 | -4.6584 |
| | Temporal_Sup_L | 20 | -54 | -12 | 9 | -5.156 |
| | Temporal_Sup_L | 11 | -48 | -27 | 6 | -3.9426 |
| | Parietal_Inf_L | 16 | -33 | -51 | 51 | -4.9971 |
| LOAD>Younger control | Frontal_Med_Orb_L | 17 | -6 | 39 | -9 | 5.5918 |

**Figure 1**

**The difference of VMHC between patients and controls (EOAD patients *vs.* young controls; LOAD patients *vs.* old controls)**

a). The EOAD patients showed significantly decreased inter-hemispheric RSFC in HP, PHG, STG and IPC. b). The LOAD patients had increased VMHC in MFG. (P<0.001, cluster size>10 voxels, uncorrected).

Note: only the left side of the image was displayed due to the symmetric template used in the analysis

a) b)

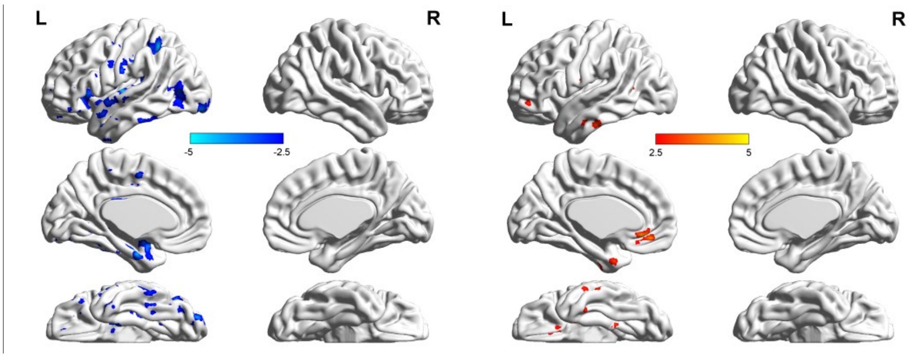


# Part 6

To show the micromotions' artifact of subjects clearly, we listed six head motion parameters below (Table 1). The two-sample t-test showed no significant difference in any index of micromotion. Moreover, we used FD as a covariate during two sample t-tests to decrease its impact on functional connectivity. We illuminated related information in the Statistical Analyses part.

**Table 1. Head motion parameters of all groups**

| | EOAD | Young controls | EOAD VS. Young controls P-value | LOAD | Old controls | LOAD VS. Old controls P-value | EOAD VS. LOAD P-value |
|---|---|---|---|---|---|---|---|
| mean(abs(Tx)) | 0.08±0.09 | 0.08±0.73 | 0.88 | 0.11±0.09 | 0.10±0.08 | 0.662 | 0.28 |
| mean(abs(Ty)) | 0.08±0.06 | 0.07±0.04 | 0.392 | 0.09±0.06 | 0.11±0.99 | 0.629 | 0.583 |
| mean(abs(Tz)) | 0.13±0.08 | 0.22±0.25 | 0.124 | 0.17±0.13 | 0.20±0.16 | 0.543 | 0.133 |
| mean(abs(Rx)) | 0.21±0.18 | 0.15±0.11 | 0.185 | 0.24±0.24 | 0.28±0.35 | 0.672 | 0.603 |
| mean(abs(Ry)) | 0.21±0.18 | 0.15±0.12 | 0.08 | 0.18±0.14 | 0.19±0.16 | 0.759 | 0.418 |
| mean(abs(Rz)) | 0.15±0.11 | 0.12±0.12 | 0.355 | 0.17±0.15 | 0.17±0.17 | 0.926 | 0.617 |
| mean FD_Power | 0.13±0.08 | 0.12±0.05 | 0.67 | 0.18±0.09 | 0.17±0.08 | 0.551 | 0.035* |

*p < 0.05, significant difference.
EOAD: early-onset Alzheimer's disease; LOAD: late-onset Alzheimer's disease;

1. Lehmann M Madison C, Ghosh PM, Miller ZA, Greicius MD, Kramer JH, Coppola G, Miller BL, Jagust WJ, Gorno-Tempini ML, Seeley WW, Rabinovici GD. Loss of functional connectivity is greater outside the default mode network in nonfamilial early-onset Alzheimer's disease variants. Neurobiol Aging. 36(10):2678-86(2015)
2. Gour N Felician O, Didic M, Koric L, Gueriot C, Chanoine V, Confort-Gouny S, Guye M, Ceccaldi M, Ranjeva JP. Functional connectivity changes differ in early and late-onset Alzheimer's disease. Hum Brain Mapp. 35(7):2978-94(2014)
